# Effects of dietary replacement of fish meal by defatted black soldier fly larvae on growth performance, blood profiles, immune response, and diarrhea incidence in weaning pigs

**DOI:** 10.5713/ab.25.0426

**Published:** 2025-10-22

**Authors:** Sooduc Noh, Xinghao Jin, Minhyuk Jang, Minsoo Park, Yooyong Kim

**Affiliations:** 1 Department of Agricultural Biotechnology and Research Institute of Agriculture and Life Sciences, Seoul National University, Seoul, Korea; 2 College of Agriculture, Yanbian University, Yanji, China

**Keywords:** Black Soldier Fly Larvae, Diarrhea Incidence, Growth Performance, Immune Response, Weaning Pigs

## Abstract

**Objective:**

The purpose of this experiment was to evaluate the effects of dietary replacement of fish meal (FM) by black soldier fly (BSF) larvae on growth performance, blood profiles, immune response, and diarrhea incidence in weaning pigs.

**Methods:**

A total of 160 weaning ([Yorkshire×Landrace]×Duroc) pigs (7.47±0.02 kg body weight [BW]) were assigned to four treatments according to sex and initial BW, with five replicates of eight pigs per pen in a randomized complete block design. Experimental diets with BSF larvae replaced FM at 0%, 25%, 50%, and 100% for phase I (0 to 2 weeks). During the phase II (3 to 4 weeks) treatments were as follows: 1) Control: corn-soybean-based diet containing FM 4%, 2) BSF25: corn-soybean-based diet containing FM 3% and BSF larvae 1%, 3) BSF50: corn-soybean-based diet containing FM 2% and BSF larvae 2%, 4) BSF100: corn-soybean-based diet containing BSF larvae 4%.

**Results:**

There were no significant differences among the treatment groups in BW and average daily gain during the experimental period. However, an increased tendency of average daily feed intake was observed (linear, p = 0.09), and gain to feed ratio tended to decrease as the replacement rate of FM with BSF larvae increased (linear, p = 0.06). During phase I, creatinine concentration decreased linearly as BSF larvae level increased (linear, p = 0.02). During phase II, the glucose concentration linearly changed as an increase in BSF larvae level (linear, p = 0.02). Meanwhile, pigs fed with increasing BSF larvae levels showed increased albumin and total protein concentration trends (linear, p = 0.05, p = 0.05).

**Conclusion:**

In weaning pig diets, defatted BSF larvae can substitute up to 50% of FM without negatively affecting immunological response, blood metabolites, or performance. These encouraging results imply that BSF larvae may be a viable and efficient substitute for FM in pig diets.

## INTRODUCTION

Global meat consumption is projected to reach about 377 million tons by 2030, a 15% increase compared with 2022, driven by population growth and economic expansion [1]. This trend will intensify the demand for feed ingredients, especially protein sources, while expansion of grain production is increasingly constrained by environmental factors. Consequently, the supply of high-quality feed proteins is expected to fall short of future demand [2]. Fish meal (FM), one of the most widely used animal-derived protein sources, faces supply risks due to ocean warming and overfishing, raising concerns about long-term sustainability [3]. Insects have recently received attention as alternative feed ingredients that can help address both nutritional and environmental challenges. Among them, black soldier fly (BSF, *Hermetia illucens*) larvae are particularly promising due to their rapid growth, minimal land requirements, high feed conversion efficiency, and ability to upcycle low-value organic materials into high-quality protein [4–6]. BSF cultivation also generates useful byproducts such as frass, which can be applied as organic fertilizer [7]. Compared with other insect species such as houseflies or mealworms, BSF is advantageous for large-scale production because adults do not feed, reducing risks of pathogen transmission [2]. Defatted BSF larvae meal typically contains 40%–50% crude protein and 7%–12% fat, with an amino acid profile comparable to FM, particularly in essential amino acids [8]. Its chitin content may also exert antimicrobial and immunomodulatory effects [9]. Recent studies have reported encouraging results when BSF larvae partially replaced conventional protein sources such as soybean meal, FM, and plasma protein in pig diets [10–12]. Beyond nutritional adequacy, BSF farming supports circular economy principles by recycling food waste into animal protein, thus reducing environmental impacts and potentially lowering production costs [13,14]. Despite these advantages, most studies have evaluated single inclusion levels or limited outcome parameters, and systematic investigations into graded replacement levels of FM with BSF in weaning pigs are still scarce. As the weaning phase is a critical period characterized by stress, digestive challenges, and immune development, it provides a suitable model to assess the feasibility of BSF inclusion. Therefore, the present study was conducted to evaluate the effects of replacing FM with defatted BSF larvae at different levels on growth performance, blood metabolites, immune response, and diarrhea incidence in weaning pigs.

## MATERIALS AND METHODS

### Experimental animals and management

A total of 160 weaning ([Yorkshire×Landrace]×Duroc) pigs (7.47±0.02 kg body weight [BW]) were assigned to four treatments in a randomized complete block design (RCBD), with five replicates of eight pigs per. Allocation was performed using the Experimental Animal Allotment Program (EAAP) [15], which ensured random distribution by sex and initial BW. All pigs were housed in slotted plastic floor pens equipped with a feeder and a nipple waterer and allowed *ad-libitum* access to feed and water throughout the experimental period (Phase I: 0–2 weeks and Phase II: 3–4 weeks). The temperature in the experimental house was maintained at 30°C in the first week, decreased by 1°C every week, and was 27°C in the last week.

### Experimental design and diet

The BSF larvae used in this study were supplied by Foodyworm. The nutritional composition of the BSF larvae meal and FM was presented in Table 1. The treatments for phase I (0–2 week) and phase II (3–4 week) were as follows: 1) Control: corn-soybean-based diet containing FM 4%, 2) BSF25: corn-soybean-based diet containing FM 3% and BSF larvae 1%, 3) BSF50: corn-soybean-based diet containing FM 2% and BSF larvae 2%, 4) BSF100: corn-soybean-based diet containing BSF larvae 4%. All nutrients in the experimental diets were formulated to meet or exceed the NRC [16] requirements for weaning pigs. The formula and chemical composition of the experimental diets were presented in Tables 2, 3.

### Growth performance

BW and feed intake data were collected at the end of each phase to calculate average daily gain (ADG), average daily feed intake (ADFI), and the gain to feed (G:F) ratio. In addition, the amount of feed eaten by all piglets was recorded each day, and waste feed in the feeder was recorded at the end of each phase.

### Blood sampling and analyses

Blood samples were taken from the jugular vein of twelve pigs with nearly average BWs in each treatment after 3 h of fasting to measure albumin (ALB), creatinine (CRE), glucose, total protein, globulin, urea, types of cholesterol, and immune response (IgA, IgG) when BW was recorded. The blood samples were centrifuged for 15 min at 1,207 ×g and 4°C (centrifuge 5810R; Eppendorf). The sera were transferred to 1.5 mL plastic tubes (serum tubes, BD vacutainer SSTTMII advance; Becton-Dickinson) and stored at −20°C until analysis. Serum IgG and IgA concentrations were analyzed by ELISA assay by the manufacturer’s protocols (ELISA Starter 87 Accessory Package, Pig IgG ELISA Quantitation Kit, Pig IgA ELISA Quantitation Kit; Bethyl). Total protein concentration (modular analytics, PE; Roche) and glucose (enzymatic kinetic assay; Roche) concentrations were analyzed using a blood analyzer. Total cholesterol (TC), high-density lipoprotein (HDL), low-density lipoprotein (LDL), ALB, and CRE were measured using spectrophotometric kits following the manufacturer’s instructions (TBA-120FR; Toshiba Medical Systems Corporation). Globulin was analyzed with the automatic analyst Olympus AU 600 (Diagnostica).

### Incidence of diarrhea

The diarrhea incidence was determined at 8:00 am for 28 days. Data were recorded by pen during phases 1 and 2. The incidence of diarrhea was scored on a 4-point scale according to the condition of the feces and diarrhea (0 = normal feces; 1 = moist feces; 2 = mild diarrhea; 3 = severe and watery diarrhea). Slightly wet feces on the rump area was designated as contaminated. After recording these data, the watery diarrhea was cleaned away.

### Statistical and chemical analyses

All the collected data were carried out using least squares mean comparisons and evaluated using SAS’s general linear model (GLM) procedure [17]. Every pen was used as one unit in the feeding trial, and the individual pig was used as an experimental unit in blood profiles, immune response, and incidence of diarrhea. Orthogonal polynomial contrasts were performed to determine linear and quadratic effects of inclusion levels of BSF larvae. A one-way ANOVA analysis was performed to compare the control to other treatments. Statistical differences were considered highly significant differences at p<0.01, significant differences at p<0.05, and tendencies between p≥0.05 and p≤0.10.

## RESULTS

### Growth performance

The effects of BSF larvae replacing FM on growth performance were presented in Table 4. There were no significant differences between the treatment groups for BW and ADG during the experimental period. However, an increased tendency of ADFI was observed (linear, p = 0.09), and G:F ratio tended to decrease as the replacement rate of FM with BSF larvae increased (linear, p = 0.06).

### Blood profiles

The effects of BSF larvae levels on blood profiles were presented in Table 5. During phase I, CRE concentration decreased linearly as BSF larvae level increased (linear, p = 0.02). During phase II, a linear response was observed in the change of glucose concentration as an increase in BSF larvae level (linear, p = 0.02). Meanwhile, pigs fed with increasing BSF larvae levels showed increased ALB and total protein concentration trends (linear, p = 0.05, p = 0.05).

### Immune response

The effects of BSF larvae levels on immune response were presented in Table 6. There were no significant differences in IgA and IgG concentrations among all treatments.

### Diarrhea incidence

The effects of BSF larvae replacing FM on the incidence of diarrhea were presented in Table 7. Pigs fed different levels of BSF larvae did not affect the incidence of diarrhea.

## DISCUSSION

The defatted BSF larvae meal used in this study contained 593.4 g/kg of crude protein (CP) and 48.3 g/kg of crude fat (CF), values comparable with FM (634.8 g/kg CP and 67.5 g/kg CF). These results are in line with previous reports, which showed that defatted BSF larvae typically contain 40.8% to 60.7% CP and 7.9% to 12.3% ether extract (EE) [18]. Although the amino acid profile of BSF larvae was generally lower than that of FM, several essential amino acids such as isoleucine, phenylalanine, proline, and serine were present in comparable amounts. In addition, BSF larvae contain chitin, a fibrous polysaccharide that can only be degraded by endogenous chitinase into chitooligosaccharides, which may serve as prebiotics to improve gut health and intestinal barrier function [19]. The present study demonstrated that replacing 4% FM with defatted BSF larvae had no detrimental effects on BW and ADG throughout the experimental phases. This finding is consistent with Driemeyer [20], who reported that partial FM replacement with BSF larvae (3.5%) did not alter BW or ADG in piglets. Similarly, Chang et al [21] confirmed that defatted and hydrolyzed BSF larvae could substitute FM at 3% of the weaning diet without negative impacts on growth. In our trial, the BW and ADG of nursery pigs up to day 21 were unaffected even when BSF larvae contributed up to 50% of animal protein. However, some studies have shown improved growth performance with insect-based diets. Jin et al [22] observed that dietary inclusion of dried mealworms (up to 6%) enhanced BW and ADG, while Lee et al [23] reported greater BW and ADG at 6 weeks when FM was completely replaced by defatted BSF larvae compared with a control containing 5% FM. These discrepancies may stem from variations in nutrient composition among insect species, differences in rearing substrate and larval age at harvest, or processing methods that affect protein digestibility and bioactive compounds [18,19]. Notably, although no statistical differences in BW or ADG were detected at the final phase, the BSF50 group exhibited numerically higher values, supporting the feasibility of FM replacement up to 50% in practical diets. In general, newly weaned pigs experience multiple concurrent stressors—including abrupt maternal separation, environmental changes, and social hierarchy establishment—which reduce feed intake, predispose to post-weaning diarrhea, and restrict growth [24]. Consequently, highly palatable and digestible ingredients are recommended in nursery diets. In line with previous reports, our data showed a tendency for increased ADFI as BSF inclusion rose, although the G:F ratio tended to decrease. Jin et al [22] reported linear improvements in both ADFI and G:F with mealworm supplementation, while Biasato et al [12] attributed greater feed intake in BSF-fed piglets to enhanced diet palatability. In our trial, the reduced G:F ratio may reflect the progressive increase in dietary chitin (0.08% in BSF25, 0.17% in BSF50, and 0.34% in BSF100). Recent evidence also suggests that high chitin intake may reduce feed efficiency by interfering with nutrient absorption and increasing endogenous losses [19]. Thus, while BSF larvae appear to support feed intake, optimizing processing methods to reduce chitin could further enhance feed efficiency in swine nutrition. In Phase II, the linear increases in ALBand total protein indicate efficient utilization of dietary protein from BSF. ALB, which represents 50%–60% of serum protein, is synthesized in the liver and serves as a reliable marker of protein supply and nutritional status [25]. The increase observed here is more likely attributable to the high protein content and digestibility of BSF rather than dehydration, since diarrhea incidence was unaffected. Consistent with our findings, previous studies also reported increased ALB and total protein in pigs receiving BSF or other insect-based diets [26,27]. The decline in CRE observed in Phase I remained within the normal physiological range for pigs (0.6–1.6 mg/dL) [28,29] and may reflect improved nitrogen utilization and reduced muscle catabolism. Recent reports further showed that insect proteins lowered serum CRE and blood urea nitrogen compared with FM-based diets, supporting this interpretation [30]. In addition, the linear rise in glucose in Phase II could reflect altered nutrient partitioning associated with the supply of highly digestible amino acids from BSF [21]. Collectively, these results suggest that BSF inclusion modulates protein and energy metabolism in a manner consistent with efficient nutrient use, without adverse effects on systemic health.

IgA is the primary immunoglobulin at mucosal surfaces, while IgG is the major antibody in serum and interstitial fluids, both crucial for host defense [25,31]. Previous studies have shown that BSF larvae can enhance immune function through gut microbiota modulation and increased mucosal IgA [27,32], largely attributed to chitin and antimicrobial peptides [33,34]. However, in this study, serum IgA and IgG were not significantly affected, consistent with reports that mealworm or defatted BSF diets did not alter systemic immunoglobulins [26]. This discrepancy may be due to the defatting process reducing bioactive components and to limited digestibility of chitin, which depends on endogenous chitinase activity [19,35]. Therefore, while BSF larvae contain immunomodulatory factors, their systemic effects may be modest under normal health conditions, and future work should focus on mucosal immunity and local gut responses. Jin et al [26] demonstrated that full-fat BSF larvae powder rich in antibacterial peptides and chitosan enhanced gut health and reduced postweaning diarrhea in piglets, and Boontiam et al [32] similarly reported decreased diarrhea when BSF larvae meal was included up to 12%. In contrast, other studies observed no effect when defatted BSF larvae replaced conventional protein sources [21,36], likely due to lower fat and bioactive components after defatting. Moreover, the limited secretion of chitin-degrading enzymes in piglets may restrict the prebiotic benefits of chitin [19], explaining why diarrhea incidence was unaffected in the present study.

## CONCLUSION

In conclusion, defatted BSF larvae can effectively replace up to 50% of FM in weaning pig diets without detrimental effects on growth performance or health parameters. These findings support the viability of BSF as a sustainable alternative protein source. Future studies should explore the long-term effects of BSF inclusion on nutrient digestibility, economic feasibility, and environmental impact across different stages of pig production.

## Figures and Tables

**Table 1:** Nutritional composition of black soldier fly (BSF) larvae and fish meal

Nutrients	BSF larvae meal	Fish meal
Moisture (%)	2.07	8.30
Crude protein (%)	59.34	63.48
Crude fat (%)	4.83	6.98
Ash (%)	19.57	19.05
Crude fiber (%)	11.19	0.21
Chitin (%)	8.42	-
Calcium (%)	6.49	6.37
Phosphorus (%)	0.98	2.97
Amino acid profile (%)		
Alanine	3.51	4.02
Arginine	2.87	3.87
Glycine	3.13	4.31
Histidine	1.77	1.52
Isoleucine	2.38	2.69
Leucine	4.02	4.70
Lysine	3.45	4.83
Phenylalanine	2.34	2.68
Proline	3.25	3.56
Serine	2.45	2.52
Threonine	2.25	2.61
Cystine	0.38	0.65
Methionine	0.96	1.71

Lab analysis values.

**Table 2:** Formula and chemical composition of the experimental diets during phase I (0–2 wk)

Items	Treatment1)			

	Control	BSF25	BSF50	BSF100
Ingredient (%)				
Expanded corn	66.37	66.11	65.79	65.47
Soybean meal	9.40	9.60	9.70	9.90
Whey	10.00	10.00	10.00	10.00
Soy oil	3.00	2.90	2.90	2.80
Plasma protein	4.00	4.00	4.00	4.00
Fish meal	4.00	3.00	2.00	0.00
Black soldier fly (BSF) larvae	0.00	1.00	2.00	4.00
L-Lysine, 98%	0.42	0.43	0.44	0.46
DL-Met, 98%	0.24	0.24	0.25	0.27
L-Threonine, 98.5%	0.18	0.18	0.18	0.18
Monocalcium phosphate	1.40	1.45	1.55	1.73
Limestone	0.00	0.10	0.20	0.20
Vit. Mix2)	0.12	0.12	0.12	0.12
Min. Mix3)	0.12	0.12	0.12	0.12
Salt	0.50	0.50	0.50	0.50
Zinc Oxide	0.25	0.25	0.25	0.25
Sum	100.00	100.00	100.00	100.00
Chemical composition4)				
Metabolizable energy (kcal/kg)	3,517.00	3,512.00	3,510.00	3,510.00
Crude protein (%)	17.60	17.63	17.60	17.59
Crude fat (%)	6.26	6.23	6.30	6.34
SID lysine (%)	1.37	1.37	1.37	1.37
SID methionine (%)	0.52	0.52	0.52	0.52
SID threonine (%)	0.90	0.90	0.89	0.89
Total calcium (%)	0.58	0.61	0.60	0.58
Total phosphorus (%)	0.79	0.79	0.80	0.80
Chitin (%)5)	0.00	0.08	0.17	0.34

1) Control: corn-soybean meal-based diet containing fish meal 4%, BSF25: corn-soybean meal based diet containing fish meal 3% and BSF larvae 1%, BSF50: corn-soybean meal based diet containing fish meal 2% and BSF larvae 2%, BSF100: corn-soybean meal based diet containing BSF 4%.

2) Contents of vitamins provided per kg of complete diet: vitamin A, 10,000 IU; vitamin D3, 2,000 IU; vitamin E, 60 IU; vitamin K, 3.5 mg; vitamin B2, 8 mg; vitamin B6, 2 mg; vitamin B12, 35 μg; pantothenic acid, 25 mg; biotin, 100 μg; niacin, 50 mg; folic acid 3.1 mg; thiamine, 1.5 mg.

3) Contents of minerals provided per kg of complete diet: Fe, 100 mg; Mn, 50 mg; Zn, 50 mg; Cu, 80 mg; Se, 400 μg; I, 1 mg.

4) Lab analysis values.

5) Calculated values based on the chitin content of BSF (8.42%).

**Table 3:** Formula of the experimental diets during phase II (3–4 wk)

Items	Treatment1)			

	Control	BSF25	BSF50	BSF100
Ingredient (%)				
Expanded corn	63.62	63.28	62.89	62.25
Soybean meal	15.80	16.00	16.20	16.40
Whey	9.00	9.00	9.00	9.00
Soy oil	2.70	2.70	2.70	2.70
Plasma protein	2.00	2.00	2.00	2.00
Fish meal	4.00	3.00	2.00	0.00
Black soldier fly (BSF) larvae	0.00	1.00	2.00	4.00
L-Lysine, 98%	0.49	0.50	0.50	0.52
DL-Met, 98%	0.30	0.31	0.31	0.33
L-Threonine, 98.5%	0.22	0.22	0.23	0.23
Monocalcium phosphate	0.60	0.62	0.70	0.90
Limestone	0.30	0.40	0.50	0.70
Vit. Mix2)	0.12	0.12	0.12	0.12
Min. Mix3)	0.10	0.10	0.10	0.10
Salt	0.50	0.50	0.50	0.50
Zinc oxide	0.25	0.25	0.25	0.25
Sum	100.00	100.00	100.00	100.00
Chemical composition4)				
Metabolizable energy (kcal/kg)	3,493.00	3,493.00	3,492.00	3,490.00
Crude protein (%)	18.70	18.73	18.74	18.69
Crude fat (%)	5.94	6.00	6.06	6.19
SID lysine (%)	1.45	1.45	1.45	1.45
SID methionine (%)	0.60	0.60	0.60	0.60
SID threonine (%)	0.94	0.94	0.94	0.94
Total calcium (%)	0.59	0.59	0.60	0.62
Total phosphorus (%)	0.61	0.60	0.60	0.60
Chitin (%)5)	0.00	0.08	0.17	0.34

1) Control: corn-soybean meal-based diet containing fish meal 4%, BSF25: corn-soybean meal based diet containing fish meal 3% and BSF larvae 1%, BSF50: corn-soybean meal based diet containing fish meal 2% and BSF larvae 2%, BSF100: corn-soybean meal based diet containing BSF 4%.

2) Contents of vitamins provided per kg of complete diet: vitamin A, 10,000 IU; vitamin D3, 2,000 IU; vitamin E, 60 IU; vitamin K, 3.5 mg; vitamin B2, 8 mg; vitamin B6, 2 mg; vitamin B12, 35 μg; pantothenic acid, 25 mg; biotin, 100 μg; niacin, 50 mg; folic acid 3.1 mg; thiamine, 1.5 mg.

3) Contents of minerals provided per kg of complete diet: Fe, 100 mg; Mn, 50 mg; Zn, 50 mg; Cu, 80 mg; Se, 400 μg; I, 1 mg.

4) Lab analysis values.

5) Calculated values based on the chitin content of BSF (8.42%).

**Table 4:** Effects of different levels of black soldier fly (BSF) larvae on growth performance in weaning pigs

Criteria	Treatment1)				SEM	p-value		
	
	Control	BSF25	BSF50	BSF100		ANOVA	Lin	Quad
Body weight (kg)								
Initial	7.47				-	-	-	-
2 wk	10.79	10.95	10.89	10.71	0.253	0.88	0.84	0.45
4 wk	16.76	16.50	16.91	16.05	0.443	0.42	0.34	0.47
ADG (g)								
0–2 wk	237.14	248.34	244.21	230.89	9.417	0.88	0.83	0.44
2–4 week	426.36	396.91	429.64	381.30	17.265	0.52	0.39	0.78
0–4 week	331.75	322.63	336.84	306.10	10.491	0.43	0.34	0.46
ADFI (g)								
0–2 wk	295.35	303.71	299.07	306.23	9.288	0.88	0.54	0.96
2–4 wk	626.54	608.29	670.80	672.00	22.423	0.30	0.16	0.48
0–4 wk	460.95	456.00	484.94	488.86	13.865	0.23	0.09	0.48
G:F ratio								
0–2 wk	0.80	0.80	0.82	0.75	0.02	0.71	0.53	0.43
2–4 wk	0.70	0.64	0.64	0.57	0.03	0.44	0.13	0.84
0–4 wk	0.73	0.69	0.69	0.63	0.02	0.21	0.06	0.54

1) Control: corn-soybean meal-based diet containing fish meal 4%, BSF25: corn-soybean meal based diet containing fish meal 3% and BSF larvae 1%, BSF50: corn-soybean meal based diet containing fish meal 2% and BSF larvae 2%, BSF100: corn-soybean meal based diet containing BSF 4%.

SEM, standard error of means; ANOVA, analysis of variance; Lin, linear; Quad, quadratic; ADG, average daily gain; ADFI, average daily feed intake; G:F, gain to feed.

**Table 5:** Effects of different levels of black soldier fly (BSF) larvae on blood profiles in weaning pigs

Criteria	Treatment1)				SEM	p-value		
	
	CON	BSF25	BSF50	BSF100		ANOVA	Lin	Quad
Albumin (g/dL)								
Initial	3.48							
Week 2	2.46	2.40	2.44	2.62	0.081	0.76	0.40	0.54
Week 4	2.44	2.36	2.42	2.80	0.081	0.15	0.05	0.20
Creatinine (mg/dL)								
Initial	0.99							
Week 2	0.77	0.73	0.67	0.53	0.677	0.22	0.02	0.77
Week 4	0.91	0.94	0.88	0.94	0.030	0.92	0.90	0.74
Glucose (mg/dL)								
Initial	109.80							
Week 2	99.00	88.60	90.00	93.40	2.952	0.65	0.71	0.29
Week 4	104.40	100.00	107.60	109.80	2.526	0.14	0.02	0.32
Total protein (g/dL)								
Initial	4.84							
Week 2	4.40	4.60	4.54	4.78	0.072	0.55	0.12	0.99
Week 4	5.20	5.14	5.08	5.78	0.123	0.10	0.05	0.13
Globulin (g/dL)								
Initial	1.36							
Week 2	1.94	2.20	2.10	2.16	0.073	0.80	0.49	0.56
Week 4	2.76	2.78	2.66	2.98	0.077	0.92	0.24	0.26
Urea (mg/dL)								
Initial	15.36							
Week 2	14.38	17.66	16.14	17.16	1.154	0.81	0.58	0.68
Week 4	21.22	20.44	18.84	21.70	0.880	0.36	0.84	0.29
Total cholesterol (mg/dL)								
Initial	98.8							
Week 2	81.80	85.40	77.80	71.60	2.397	0.51	0.09	0.59
Week 4	101.80	110.60	110.00	109.80	4.530	0.89	0.63	0.60
HDL cholesterol (mg/dL)								
Initial	42.00							
Week 2	35.60	32.40	34.80	28.20	1.309	0.62	0.10	0.61
Week 4	35.80	40.00	40.80	38.60	1.603	0.71	0.65	0.30
LDL cholesterol (mg/dL)								
Initial	51.80							
Week 2	43.60	51.20	41.40	40.80	1.705	0.21	0.19	0.49
Week 4	63.60	68.60	67.00	69.60	4.351	0.97	0.71	0.88

1) Control: corn-soybean meal-based diet containing fish meal 4%, BSF25: corn-soybean meal based diet containing fish meal 3% and BSF larvae 1%, BSF50: corn-soybean meal based diet containing fish meal 2% and BSF larvae 2%, BSF100: corn-soybean meal based diet containing BSF 4%.

SEM, standard error of means; ANOVA, analysis of variance; Lin, linear; Quad, quadratic; HDL, high-density lipoprotein; LDL, low-density lipoprotein.

**Table 6:** Effects of different levels of black soldier fly (BSF) larvae on immune response in weaning pigs

Criteria	Treatment1)				SEM	p-value		
	
	Control	BSF25	BSF50	BSF100		ANOVA	Lin	Quad
IgA (mg/mL)								
Initial	0.52							
2 wk	0.62	0.55	0.62	0.54	0.038	0.48	0.56	0.48
4 wk	0.60	0.57	0.56	0.59	0.016	0.32	0.52	0.32
IgG (mg/mL)								
Initial	4.89							
2 wk	4.85	5.36	5.01	4.17	0.432	0.23	0.46	0.25
4 wk	5.39	5.65	6.03	5.58	0.233	0.25	0.11	0.15

1) Control: corn-soybean meal-based diet, BSF25: corn-soybean meal based diet with 25% of plasma protein replaced by black soldier fly larvae, BSF50: corn-soybean meal based diet with 50% of plasma protein replaced by black soldier fly larvae, BSF100: corn-soybean meal based diet with 100% of plasma protein replaced by black soldier fly larvae.

SEM, standard error of means; ANOVA, analysis of variance; Lin, linear; Quad, quadratic.

**Table 7:** Effects of different levels of black soldier fly (BSF) larvae on diarrhea incidence in weaning pigs

Criteria	Treatment1)				SEM	p-value		
	
	Control	BSF25	BSF50	BSF100		ANOVA	Lin	Quad
Diarrhea incidence								
2 wk	2.11	2.12	2.14	2.06	0.032	0.73	0.47	0.45
4 wk	0.39	0.43	0.42	0.38	0.177	0.72	0.80	0.33
0–4 wk	0.72	0.74	0.74	0.71	0.013	0.64	0.59	0.25

1) Control: corn-soybean meal-based diet, BSF25: corn-soybean meal based diet with 25% of plasma protein replaced by black soldier fly larvae, BSF50: corn-soybean meal based diet with 50% of plasma protein replaced by black soldier fly larvae, BSF100: corn-soybean meal based diet with 100% of plasma protein replaced by black soldier fly larvae.

SEM, standard error of means; ANOVA, analysis of variance; Lin, linear; Quad, quadratic.

## Data Availability

Upon reasonable request, the datasets of this study can be available from the corresponding author.

## References

[1] Organisation for Economic Co-operation and Development (OECD), Food and Agriculture Organization of the United Nations (FAO) (2022). OECD-FAO agricultural outlook 2022–2031.

[2] DiGiacomo K, Leury BJ (2019). Review: insect meal: a future source of protein feed for pigs?. Animal.

[3] Ha SS, Kim KJ (2018). A study on climate variability and its impact on anchoveta landing, correlation of fish meal production and price in Peru. Lat Am Caribb Stud.

[4] Van Huis A, Van Itterbeeck J, Klunder H (2013). Edible insects: future prospects for food and feed security.

[5] van Huis A (2013). Potential of insects as food and feed in assuring food security. Annu Rev Entomol.

[6] Regina M, Murta D, Metin T, Yildirim E (2021). The insects as a workforce for organic fertilizers production: insect frass. New generation of organic fertilizers.

[7] Nyakeri EM, Ogola HJ, Ayieko MA, Amimo FA (2017). An open system for farming black soldier fly larvae as a source of proteins for smallscale poultry and fish production. J Insects Food Feed.

[8] Elwert C, Knips I, Katz P (2010). A novel protein source: maggot meal of the black soldier fly (Hermetia illucens) in broiler feed. Tagung Schweine-und Geflügelernährung.

[9] Abdel-Ghany HM, Salem MES (2020). Effects of dietary chitosan supplementation on farmed fish; a review. Rev Aquac.

[10] Neumann C, Velten S, Liebert F (2018). N balance studies emphasize the superior protein quality of pig diets at high inclusion level of algae meal (Spirulina platensis) or insect meal (Hermetia illucens) when adequate amino acid supplementation is ensured. Animals.

[11] Altmann BA, Neumann C, Rothstein S, Liebert F, Mörlein D (2019). Do dietary soy alternatives lead to pork quality improvements or drawbacks? A look into micro-alga and insect protein in swine diets. Meat Sci.

[12] Biasato I, Renna M, Gai F (2019). Partially defatted black soldier fly larva meal inclusion in piglet diets: effects on the growth performance, nutrient digestibility, blood profile, gut morphology and histological features. J Anim Sci Biotechnol.

[13] van Huis A (2021). Advancing edible insects as food and feed in a circular economy. J Insects Food Feed.

[14] van Huis A (2020). Insects as food and feed, a new emerging agricultural sector: a review. J Insects Food Feed.

[15] Kim BG, Lindemann MD (2007). A new spreadsheet method for the experimental animal allotment. J Anim Sci.

[16] Committee on Nutrient Requirements of Swine (2012). National Research Council Nutrient requirements of swine.

[17] SAS Institute (2004). SAS user’s guide: statistics. ver. 7.0.

[18] Hong J, Kim YY (2022). Insect as feed ingredients for pigs. Anim Biosci.

[19] Liu S, Wang J, Li L (2023). Endogenous chitinase might lead to differences in growth performance and intestinal health of piglets fed different levels of black soldier fly larva meal. Anim Nutr.

[20] Driemeyer H (2016). Evaluation of black soldier fly (Hermetia illucens) larvae as an alternative protein source in pig creep diets in relation to production, blood and manure microbiology parameters [master’s thesis].

[21] Chang SY, Kim KH, Lee BK (2024). Defatted or hydrolyzed black soldier fly larvae have sufficient potential as an alternative to fishmeal for weaned pigs. Anim Feed Sci Technol.

[22] Jin XH, Heo PS, Hong JS, Kim NJ, Kim YY (2016). Supplementation of dried mealworm (Tenebrio molitor larva) on growth performance, nutrient digestibility and blood profiles in weaning pigs. Asian-Australas J Anim Sci.

[23] Lee J, Park Y, Song D, Chang S, Cho J (2024). Effects of defatted and hydrolyzed black soldier fly larvae meal as an alternative fish meal in weaning pigs. Animals.

[24] Tang X, Xiong K, Fang R, Li M (2022). Weaning stress and intestinal health of piglets: a review. Front Immunol.

[25] Rosenoer VM, Oratz M, Rothschild MA (2014). Albumin: structure, function and uses.

[26] Jin X, Yuan B, Liu M (2021). Dietary Hermetia illucens larvae replacement alleviates diarrhea and improves intestinal barrier function in weaned piglets challenged with enterotoxigenic Escherichia coli K88. Front Vet Sci.

[27] Yu M, Li Z, Chen W, Rong T, Wang G, Ma X (2019). Hermetia illucens larvae as a potential dietary protein source altered the microbiota and modulated mucosal immune status in the colon of finishing pigs. J Anim Sci Biotechnol.

[28] Ostermann M, Kashani K, Forni LG (2016). The two sides of creatinine: both as bad as each other?. J Thorac Dis.

[29] Iwase H, Yamamoto T, Cooper DKC (2018). Episodes of hypovolemia/dehydration in baboons with pig kidney transplants: a new syndrome of clinical importance?. Xenotransplantation.

[30] Adebiyi FG, Dunmade AS, David PE (2024). Effects of partially defatted black soldier fly larvae (Hermetia illucens) based diets on growth performance, nutrient digestibility, and serum biochemistry profile of weaned pigs. Niger Agric J.

[31] Senay LC, Christensen ML (1965). Changes in blood plasma during progressive dehydration. J Appl Physiol.

[32] Boontiam W, Phaengphairee P, Hong J, Kim YY (2022). Full-fatted Hermetia illucens larva as a protein alternative: effects on weaning pig growth performance, gut health, and antioxidant status under poor sanitary conditions. J Appl Anim Res.

[33] Elieh Ali Komi D, Sharma L, Dela Cruz CS (2018). Chitin and its effects on inflammatory and immune responses. Clin Rev Allergy Immunol.

[34] Xia J, Ge C, Yao H (2021). Antimicrobial peptides from black soldier fly (Hermetia illucens) as potential antimicrobial factors representing an alternative to antibiotics in livestock farming. Animals.

[35] Xing R, Liu Y, Li K (2017). Monomer composition of chitooligosaccharides obtained by different degradation methods and their effects on immunomodulatory activities. Carbohydr Polym.

[36] Choi YH, Kim JE, Jung HJ, Cho ES, Kim DW, Kim JS (2020). Effects of Hermetia illucens supplementation on fecal score, blood profiles, immune response and small intestinal morphology in weaned pigs. J Korea Acad Ind Coop Soc.

